# Global variations in pubertal growth spurts in adolescents living with perinatal HIV

**DOI:** 10.1097/QAD.0000000000003602

**Published:** 2023-05-17

**Keywords:** adolescents, antiretroviral therapy, growth, HIV, perinatal, puberty

## Abstract

To describe pubertal growth spurts among adolescents living with perinatally acquired HIV (ALWPHIV) on antiretroviral therapy (ART).

**Design:**

Observational data collected from 1994 to 2015 in the CIPHER global cohort collaboration.

**Methods:**

ALWPHIV who initiated ART age less than 10 years with at least four height measurements age at least 8 years were included. Super Imposition by Translation And Rotation (SITAR) models, with parameters representing timing and intensity of the growth spurt, were used to describe growth, separately by sex. Associations between region, ART regimen, age, height-for-age (HAZ), and BMI-for-age *z*-scores (BMIz) at ART initiation (baseline) and age 10 years, and SITAR parameters were explored.

**Results:**

Four thousand seven hundred and twenty-three ALWPHIV were included: 51% from East and Southern Africa (excluding Botswana and South Africa), 17% Botswana and South Africa, 6% West and Central Africa, 11% Europe and North America, 11% Asia-Pacific, and 4% Central, South America, and Caribbean. Growth spurts were later and least intense in sub-Saharan regions. In females, older baseline age and lower BMIz at baseline were associated with later and more intense growth spurts; lower HAZ was associated with later growth spurts. In males, older baseline age and lower HAZ were associated with later and less intense growth spurts; however, associations between baseline HAZ and timing varied by age. Lower HAZ and BMIz at 10 years were associated with later and less intense growth spurts in both sexes.

**Conclusion:**

ALWPHIV who started ART at older ages or already stunted were more likely to have delayed pubertal growth spurts. Longer-term follow-up is important to understand the impact of delayed growth.

## Introduction

With increasing availability of antiretroviral therapy (ART), there are increasing numbers of children with perinatally acquired HIV living into adolescence and adulthood [1]. Globally, in 2020, there were an estimated 2.78 million young people aged 0–19 years living with HIV, including 1.75 million adolescents aged 10–19 years, most in sub-Saharan Africa [2]. Although immediate ART is now recommended for all children living with HIV [3], for the current population of ALWPHIV access to ART is likely to have been delayed, and globally only 54% of 10–19-year-old ALWPHIV were on ART in 2020 [2].

Adolescence is a critical period for growth, and may offer a potential window in which earlier growth deficiencies may, in part, be corrected [4,5]. The adolescent growth spurt is a period of rapid growth during which ∼15% of final adult height and 45% of maximum skeletal mass is gained [6] with the highest peak growth velocity generally observed in those with earlier puberty [7]. ALWPHIV often experience later puberty than young people HIV-exposed uninfected [8,9] reaching lower final heights [10]. A US study found much of the difference in age at sexual maturity between ALWPHIV and young people HIV-exposed uninfected can be explained by poorer growth among the ALWPHIV earlier in life [9]. Despite potential catch-up growth after starting ART, some studies have observed delays in pubertal development associated with growth deficits present at ART initiation, irrespective of age at ART initiation [11,12].

Growth deficits and delayed puberty can affect the psychological wellbeing of ALWPHIV, with lower height associated with increased symptoms of depression [13]. There may also be a lasting impact on future physical health. As a result of HIV infection and lifelong exposure to ART, ALWPHIV are at increased risk of low bone mineral density, and delays in pubertal onset may increase the risk of poor bone health and in turn increase the risk of fractures and osteoporosis [14].

The Collaborative Initiative for Paediatric HIV Education and Research (CIPHER) global cohort collaboration recently described the evolution of height-for-age *z*-scores (HAZ) in ALWPHIV who initiated ART by age 10 years, highlighting global variations [15] with patterns seen in sub-Saharan Africa indicative of delayed pubertal growth spurts. The aim of this study was to complement previous work by describing characteristics associated with the timing and intensity of pubertal growth spurts in ALWPHIV.

## Methods

Individual patient-level data collected from 1994 to 2015 were pooled from 11 paediatric HIV cohort networks: Baylor International Pediatric AIDS Initiative (BIPAI); European Pregnancy and Paediatric Infections Cohort Collaboration (EPPICC); the IeDEA Consortium, constituting IeDEA Asia-Pacific, IeDEA Central Africa, IeDEA East Africa, IeDEA Southern Africa, IeDEA West Africa, and Central and South America and the Caribbean network for HIV epidemiology (CCASAnet); International Maternal Pediatric Adolescent AIDS Clinical Trials (IMPAACT) 219C and P1074; Optimal Models (ICAP at Columbia University); and the Pediatric HIV/AIDS Cohort Study (PHACS). The data represent a range of care settings, including dedicated research cohorts and programmatic services. There was no overlap in the coverage of the networks, therefore, no duplication of participants amongst networks. The epidemiology of ALWPHIV in CIPHER has previously been described [16].

Each participating network obtained ethics approval from their respective institutional review boards to contribute data. Consent or assent requirements were according to local institutional review board requirements. All analyses were prespecified and approved by the CIPHER Project Oversight Group.

Adolescents known to have acquired HIV perinatally or who were aged less than 10 years at first presentation to care (proxy for perinatally acquired HIV) and initiated ART age less than 10 years on a ‘standard’ combination regimen, defined as a nonnucleoside reverse transcriptase inhibitor (NNRTI) plus at least two nucleoside/nucleotide reverse transcriptase inhibitors (NRTI), boosted protease inhibitor (PI) plus at least two NRTI, or a three NRTI regimen including abacavir, were eligible for this study. Those included in analysis were required to have height and weight reported at ART initiation and at least four height measurements on or after their eighth birthday, including at least one measurement at age 12 years or older for female individuals and at 14 years or older for male individuals (assuming, on average, peak height velocity will occur before age 12 in female individuals and before age 14 in male individuals [7]).

Height, weight and BMI at ART initiation (closest within 6 months before to 1 month after) and age 10 years (closest within 6 months) were converted to HAZ, weight-for-age *z*-scores (WAZ) and BMI-for-age *z*-scores (BMIz), using WHO child growth standards [17] and the WHO 2007 growth reference [18].

Countries were grouped into geographical regions based on categorizations used by UNAIDS [2]. Three sub-Saharan African regions were included. As Botswana and South Africa are both upper middle-income countries, they were grouped separately from other Southern African countries. Previous analyses demonstrated differential growth patterns in East and Southern Africa (excluding Botswana and South Africa) compared with West and Central Africa [15]. Furthermore, this study included data through 2015, at which time ART access in West and Central Africa lagged behind East and Southern Africa [19]. Final groupings were: North America and Europe; Central and South America, and the Caribbean; Asia-Pacific; West and Central Africa; Botswana and South Africa; and East and Southern Africa (excluding Botswana and South Africa).

### Statistical analysis

Linear growth was modelled using Super Imposition by Translation And Rotation (SITAR) models [20]. SITAR quantifies differences in growth of each individual from the population average growth curve via three parameters that represent average height (larger values indicate taller height throughout adolescence), timing (larger values indicate later growth spurts) and intensity (large values indicate steeper, more rapid, growth spurts with higher growth velocity) of the adolescent growth spurt. Further details of the SITAR model are available in Supplement S1.

Analyses were conducted separately in male and female individuals. First, SITAR models were fitted separately within each region to estimate mean regional growth curves. To further explore variations by region and characteristics at ART initiation, SITAR models were fitted including adolescents from all regions combined. The SITAR estimates of the timing and intensity of the growth spurt of each individual included in the analysis were used as dependent variables in multivariable linear regression models. In the main analysis, the independent variables included were region, age, HAZ and BMIz at ART initiation, initial ART regimen drug class and year of birth. Modelling was then repeated with HAZ and BMIz at age 10 years (+/−6 months) used in place of HAZ and BMIz at ART initiation.

For continuous independent variables, best fitting fractional polynomials were identified using the algorithm described by Royston and Sauerbrei [21]. Interactions were considered between region and other independent variables, between age at ART initiation and HAZ, between age and BMIz, and between HAZ and BMIz. Interaction terms with likelihood ratio test *P* less than 0.05 were included. SITAR models were fitted using the SITAR package v1.1.2 [22] in R v4.0.3 [23]. All other analyses were conducted using Stata IC v16.1.

## Results

**Fig. 1:**
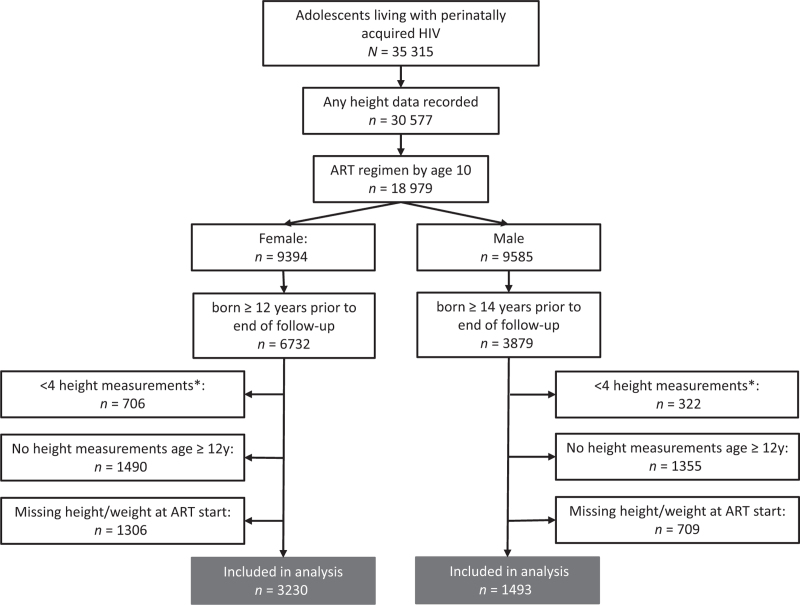
Flow diagram of selection of the adolescents living with HIV included in the puberty growth spurt analysis.

Among 35 315 ALWPHIV ever in follow-up, 18 979 (9394 female individuals) had any height data and had initiated ART by age 10 years (Fig. 1). Of 6732 female individuals born at least 12 years prior to the end of follow-up; 3230 (48%) had at least four height measurements recorded and complete data on characteristics at ART initiation, and thus were included in analysis. Of 3879 male individulas born at least 14 years prior to the end of follow-up, 1493 (38%) were included. Among the 4723 ALWPHIV included, 4020 (85%) were in active paediatric follow-up at participating clinics at data cut-off, 506 (11%) had transferred to another clinic (including adult care), 124 (3%) were lost to follow-up, 25 (1%) had dropped out for other reasons and 48 (1%) had died. Two thousand, four-hundred and ten (51%) were from East and Southern Africa, 816 (17%) Botswana and South Africa, 311 (7%) West and Central Africa, 505 (11%) Europe and North America, 502 (11%) Asia-Pacific and 179 (4%) from Central and South America and the Caribbean. Exclusions from modelling due to insufficient height data or missing characteristics at ART initiation varied by region and were most likely in East & Southern Africa (excluding Botswana & South Africa) and least likely Asia-Pacific (Supplementary Tables S1 and S2). ALWPHIV excluded were born slightly more recently (and less likely to have height data in later adolescence) and were slightly younger at ART initiation than those who were included, with these differences most pronounced in Europe and North America (Supplementary Tables S1 and S2).

CIPHER global cohort collaboration. ^∗^For inclusion in modelling at least four height measurements between age 8 and under 19 years were required. Additionally, at least one measurement at age 12 years or older was required for females and age 14 years or older for males, along with height/weight at ART start.

Among the 3230 female and 1493 male individuals included, there were 94 009 and 52 029 height measurements recorded respectively, with a median 27 [interquartile range (IQR) 18–38] per female individual and 33 (IQR 23–44) per male. Median age at latest height measurement was 13.9 (IQR 12.8–15.2) years for female individuals and 15.3 (IQR 14.6–16.4) years for male individuals, though there was some variation by region (Supplementary Table S3).

Median age at ART initiation was 7.7 (IQR 6.0–8.9) years in female individuals and 8.0 (IQR 6.6–9.1) years in male individuals; adolescents from the sub-Saharan regions started ART at older ages than in other regions (Table 1). Median HAZ, WAZ and BMIz at ART initiation varied by region, and all were higher in Europe and North America than elsewhere; HAZ and WAZ was lowest in Asia-Pacific (Table 1).

**Table 1:** Study characteristics of participants included in the analysis stratified by sex.

	All (*n* = 4723)	East and Southern Africa (excluding Botswana and South Africa) (*n* = 2410)	Botswana and South Africa (*n* = 816)	West and Central Africa (*n* = 311)	Europe and North America (*n* = 505)	Asia-Pacific (*n* = 502)	Central and South America, and the Caribbean (*n* = 179)
	Median (IQR) or *n* (%)						
Females							
Total	3230	1706 (53%)	553 (17%)	211 (7%)	297 (9%)	342 (11%)	121 (4%)
Year of birth	1999 (1997–2000)	1999 (1998–2000)	1998 (1997–2001)	1998 (1997–1999)	1997 (1995–1999)	1999 (1998–2000)	1998 (1997–2000)
At ART initiation							
Calendar year	2006 (2005–2008)	2007 (2006–2008)	2005 (2004–2007)	2005 (2005–2007)	2001 (1999–2004)	2005 (2003–2007)	2003 (2001–2004)
Age (years)	7.7 (6.0–8.9)	8.1 (6.8–9.1)	7.8 (5.8–8.8)	8.0 (6.6–9.0)	5.3 (2.4–7.8)	6.5 (5.0–7.9)	4.1 (1.7–7.1)
First regimen							
PI + ≥ 2NRTI	266 (8%)	23 (1%)	29 (5%)	17 (8%)	143 (48%)	4 (1%)	50 (41%)
NNRTI + ≥2 NRTI	2939 (91%)	1,671 (98%)	524 (95%)	194 (92%)	143 (48%)	336 (98%)	71 (59%)
3 NRTIs including abacavir	25 (<1%)	12 (<1%)	0	0	11 (4%)	2 (<1%)	0
Height-for-age *z*-score	−2.0 (−2.9 to −1.1)	−2.1 (−3.0 to −1.2)	−2.0 (−2.7 to −1.2)	−1.6 (−2.6 to −0.6)	−1.0 (−1.7 to 0.0)	−2.3 (−3.3 to −1.5)	−1.8 (−2.7 to −0.9)
Weight-for-age *z*-score	−1.7 (−2.7 to −0.8)	−1.8 (−2.8 to −1.0)	−1.6 (−2.4 to −0.8)	−1.9 (−3.1 to −0.9)	−0.5 (−1.2 to 0.3)	−2.1 (−3.3 to −1.1)	−1.3 (−2.4 to −0.5)
BMI-for-age *z*-score	−0.6 (−1.5 to 0.1)	−0.8 (−1.7 to 0.1)	−0.5 (−1.3 to 0.2)	−1.3 (−2.2 to −0.4)	0.1 (−0.6 to 0.9)	−0.9 (−1.7 to −0.1)	−0.4 (−1.2 to 0.6)
Males							
Total	1493	704 (47%)	263 (18%)	100 (7%)	208 (14%)	160 (11%)	58 (4%)
Year of birth	1998 (1996–1999)	1998 (1997–1999)	1997 (1996–1999)	1997 (1996–1998)	1996 (1993–1997)	1998 (1997–1999)	1998 (1996–1998)
At ART initiation							
Calendar year	2005 (2004–2006)	2006 (2005–2007)	2005 (2004–2006)	2005 (2005–2006)	2000 (1998–2003)	2005 (2003–2006)	2002 (2001–2004)
Age (years)	8.0 (6.6–9.1)	8.4 (7.4–9.3)	8.1 (6.4–9.1)	8.6 (7.6–9.4)	5.7 (3.2–8.0)	7.1 (5.9–8.2)	5.7 (4.0–7.5)
First regimen							
PI + ≥2 NRTI	182 (12%)	27 (4%)	13 (5%)	14 (14%)	108 (52%)	1 (<1%)	19 (33%)
NNRTI + ≥2 NRTI	1293 (87%)	674 (96%)	250 (95%)	86 (86%)	88 (44%)	159 (99%)	36 (62%)
3 NRTIs including abacavir	18 (1%)	3 (<1%)	0	0	12 (6%)	0	3 (5%)
Height-for-age *z*-score	−1.8 (−2.8 to −1.0)	−2.1 (−2.9 to −1.2)	−1.8 (−2.7 to −1.1)	−1.6 (−2.7 to −0.8)	−0.8 (−1.4 to 0.1)	−2.5 (−3.3 to −1.6)	−1.8 (−2.7 to −1.2)
Weight-for-age *z*-score	−1.8 (−2.8 to −0.8)	−2.0 (−3.0 to −1.2)	−1.6 (−2.5 to −0.9)	−2.0 (−2.8 to −1.2)	−0.2 (−1.0 to 0.6)	−2.5 (−3.5 to −1.4)	−1.5 (−2.6 to −0.6)
BMI-for-age *z*-score	−0.7 (−1.6 to 0.1)	−0.8 (−1.8 to 0.0)	−0.7 (−1.4 to 0.2)	−1.5 (−2.2 to −0.7)	0.3 (−0.5 to 1.1)	−1.0 (−1.9 to −0.1)	−0.3 (−1.3 to 0.3)

ART, antiretroviral therapy; NNRTI, nonnucleoside reverse transcriptase inhibitors; NRTI, nucleoside/nucleotide reverse transcriptase inhibitors; PI, boosted protease inhibitor.

**Fig. 2:**
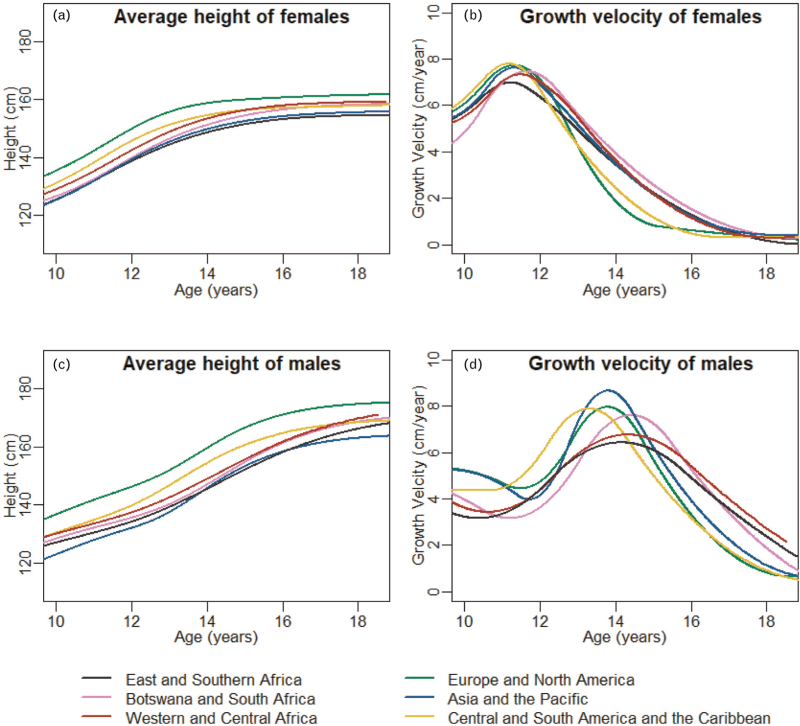
Mean height (left panel) and growth velocity (right panel) curves estimated using SITAR models stratified by region and sex in the CIPHER global cohort collaboration.

Average growth curves, estimated using SITAR models fitted separately to each region, are shown in Fig. 2. SITAR models fitted on the complete dataset (with all regions combined) explained 98% of the variation in individual growth trajectories of both male and female individuals (Supplementary Figure S1). SITAR parameters from these models were compared across the six regions (Supplementary Table S4). On average, throughout adolescence, female (Fig. 2a) and male individuals (Fig. 2c) in Europe and North America were consistently tallest and those from East and Southern Africa and Asia-Pacific shortest. In female individuals, the growth spurt was earliest and had greater intensity in Europe and North America and Central, South America and the Caribbean (Fig. 2b). Compared with East and Southern Africa, the growth spurt occurred 0.37 (95% CI 0.21–0.54) years earlier in Europe and North America, and 0.31 (0.06–0.56) years earlier in Central, South America and the Caribbean. There were also differences between sub-Saharan regions with the growth spurt occurring on average 0.43 (0.31–0.56) years later in Botswana and South Africa and 0.22 (0.03–0.41) years later in West and Central Africa compared with the rest of East and Southern Africa; though growth spurts in West and Central Africa were more intense than in East and Southern Africa. In male individuals, the growth spurt occurred at a similar time in the three African regions but with slightly greater intensity in Botswana and South Africa (Fig. 2d). Compared with East and Southern Africa, the growth spurt was 0.95 (0.45–1.45) years earlier in Central and South America, and the Caribbean, 0.49 (0.21–0.78) years earlier in Europe and North America, and 0.34 (0.02–0.66) years earlier in Asia-Pacific, while the intensity was greatest in Asia-Pacific followed by Europe and North America.

**Fig. 3:**
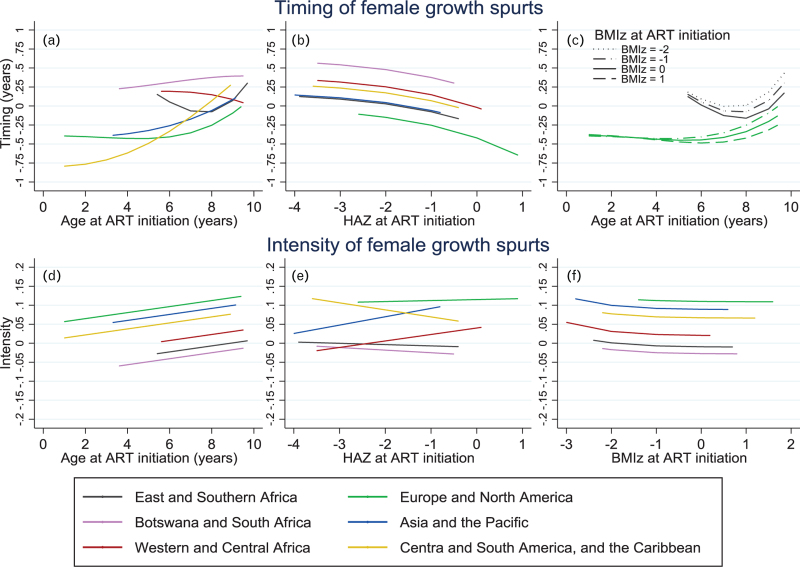
Female pubertal growth: multivariable associations between region, age, height-for-age *z*-score and BMI-for-age *z*-score at antiretroviral therapy initiation and the timing and intensity of the pubertal growth spurt in females in the CIPHER global cohort collaboration.

**Fig. 4:**
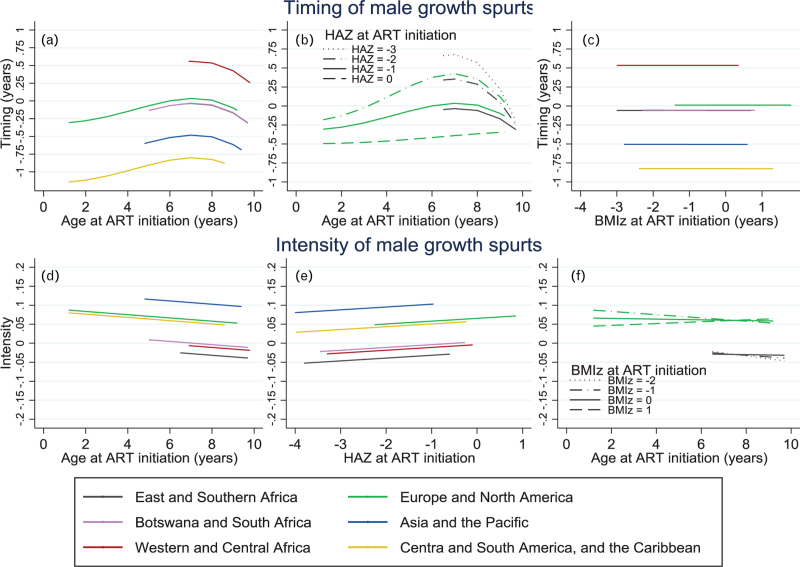
Male pubertal growth: Multivariable associations between region, age, height-for-age *z*-score and BMI-for-age *z*-score at antiretroviral therapy initiation and the timing and intensity of the pubertal growth spurt in males in the CIPHER global cohort collaboration.

Regional variations remained after adjustment in both male and female individuals (Figs. 3 and 4, see figure footnote for interpretation of figures and supplementary Tables S5–S8 for full model details). Female individuals starting ART later in childhood had later growth spurts than those who started ART at a young age, though there was some regional variation in the association between age and timing (*P* < 0.001 for interaction, Fig. 3a). Lower HAZ (*P* < 0.001, Fig. 3b) and lower BMIz (Fig. 3c) at ART initiation were associated with later growth spurts, though for BMIz, this was only seen in children starting ART at older ages (*P* = 0.025 for interaction). Older age at ART initiation (*P* < 0.001, Fig. 3d) and starting ART with very low BMIz (*P* < 0.001, Fig. 3f) were associated with a growth spurt of greater intensity. The association between HAZ at ART start and the intensity of the growth spurt varied by region (*P* < 0.001 for interaction) though there was no clear pattern (Fig. 3e).

On the y-axes, 0 represents the average time (a)–(c) and average intensity (d)–(f) of the growth spurt across all females in the analysis. Lower values represent earlier/less intense growth spurts and higher values later/more intense growth spurts. For example, (a) demonstrates that increasing age at ART is associated with later growth spurts, though there is some variation by region; (d) shows that increasing age at ART initiation is associated with more intense growth spurts. Lines represent differences in timing/intensity across regions for adolescents born in 1999, who initiated an NNRTI regimen, with a BMIz of −1 [in (a), (b), (d) and (e)] HAZ of −1 [(a), (c), (d), (f)] at age 8 years [(b), (e), (f)]. Lines are restricted to the 10th-90th percentile of age, HAZ and BMIz observed in each region. The interaction between BMIz and age for timing (c) is illustrated for adolescents in East and Southern Africa and Europe and North America. ART, antiretroviral therapy; BMIz, body mass index-for-age z-score; HAZ, height-for-age z-score; NNRTI, non-nucleoside reverse transcriptase inhibitors.

On the *y*-axes, 0 represents the average time (a)–(c) and average intensity (d)–(f) of the growth spurt across all males in the analysis. Lower values represent earlier/less intense growth spurts and higher values later/more intense growth spurts. For example, (a) demonstrates that increasing age at ART is associated with later growth spurts up to around age 7, and earlier growth spurts after age 7 years. (d) shows that increasing age at ART initiation is associated with more intense growth spurts. Lines represent differences in timing/intensity across regions for males born in 1998, who initiated an NNRTI regimen, with a BMIz of −1 [in (a), (b), (d), (e)], HAZ of −1 [(a), (c), (d), (f)] at age 8 years [(b), (c), (e)]. Lines are restricted to the 10th–90th percentile of age, HAZ and BMIz observed in each region. The interactions between HAZ and age for timing (b) and BMIz and age for intensity (f) are illustrated for adolescents in East and Southern Africa and Europe and North America. ART, antiretroviral therapy; BMIz, body mass index-for-age *z*-score; HAZ, height-for-age *z*-score; NNRTI, non-nucleoside reverse transcriptase inhibitors.

For male individuals (Fig. 4), older age at ART initiation was associated with later growth spurts for those initiating ART up to 8 years of age; those starting ART at age 8–10 years had earlier growth spurts than those starting shortly before age 8 (Fig. 4a). Lower HAZ at ART initiation was also associated with later growth spurts, though differences by HAZ varied by age at ART start (*P* = 0.039 for interaction, Fig. 4b). In male individuals who started ART with a HAZ of 0, there was little variation in timing by age at ART initiation (Fig. 4b). There was no difference in timing of growth spurts by BMIz at ART initiation (*P* = 0.99, Fig. 4c). Lower HAZ at ART initiation (*P* = 0.046, Fig. 4e) and, in those starting ART later in childhood with, lower BMIz at ART initiation (*P* = 0.028 for interaction, Fig. 4f) were associated with lower intensity during the growth spurt. Drug class of initial ART regimen was not associated with timing or intensity in male or female individuals.

In additional analyses, lower age and BMIz at age 10 years were associated with later and less intense growth spurts in both female individuals (Supplementary Figure S2 and Tables S9 and S10) and male individuals (Supplementary Figure S3 and Tables S11 and S12), irrespective of age at ART initiation.

## Discussion

This collaborative individual patient meta-analysis is the first report on the timing and intensity of the pubertal growth spurt among adolescents living with perinatally acquired HIV globally. We found growth spurts in sub-Saharan African regions were later and had lower intensity than elsewhere. These differences were present after adjusting for age, HAZ and BMIz at ART initiation, initial ART drug class and year of birth.

In line with other studies [11,12,24], growth spurts tended to occur later in those starting ART at older ages, though we observed differences between male and female individuals. In female individuals, older age at ART initiation was associated with later and more intense growth spurts, though this was less pronounced in the sub-Saharan regions than elsewhere. In male individuals, the association was weaker than that observed in female individuals, with older age at ART associated with later male growth spurts in those starting ART by age 8, but earlier growth spurts seen in those starting ART between 8 and 10 years of age. The association between age and timing of growth spurts also varied by HAZ at ART initiation; in male individuals with ‘normal’ HAZ (HAZ = 0) at ART initiation, there was little variation in timing by age at ART initiation. Lower HAZ at ART initiation was also associated with later female growth spurts, with no evidence that this differed by age at ART initiation. The patterns observed in male individuals who were older at ART initiation may reflect difficulties in distinguishing between catch-up growth on ART and rapid growth during puberty. However, similar trends were observed in Ugandan and Zimbabwean adolescents living with HIV in the ARROW trial, where older age at start of ART was associated with pubertal delay with the delay more pronounced in female individuals than in male individuals [12]. For female individuals, the pubertal delay associated with each additional year of age at ART start was greater in those who did not start ART until later childhood than in those who started in early childhood. In contrast, in male individuals, the effect of age at ART start weakened in older age.

In female individuals starting ART at older ages, lower BMIz at ART initiation was associated with later growth spurts, whereas a very low BMIz (less than –2) was associated with growth spurts of higher intensity, though numbers with BMIz less than −2 were small. For male individuals, there was no difference in timing of growth spurts by BMIz at ART initiation, but growth spurts had slightly lower intensity for those starting ART later in childhood with lower BMIz. As all ALWPHIV included in the analysis, initiated ART by age 10 years, differences between sexes may be driven by the fact that the growth spurt occurs later in male individuals. This may have allowed some effects of low zBMI at ART initiation to be reversed in male individuals before the period of peak growth velocity. In both sexes, lower BMIz (and HAZ) at age 10 years was associated with later and less intense growth spurts, irrespective of age at ART initiation. Other cohort studies in the general population have also observed sex-based differences, with an inverse relationship between BMI and age at puberty consistently observed in female individuals but not in male individuals [25].

There have been mixed findings from studies comparing catch-up growth on different ART regimens or classes, with some trials suggesting poorer growth on lopinavir/ritonavir [26–28] and others showing no differences [29,30]. We found no evidence of a difference in growth during adolescence among those who had initiated protease inhibitor versus NNRTI-based regimens. However, the majority of children initiating protease inhibitor-based regimens were from outside sub-Saharan Africa. Given the differences between initial ART by region, we may not have been adequately powered to detect a true difference.

Growth during adolescence may be affected by repeated infections, nutritional deficits, and suboptimal growth early in life [5]. Differences in timing of puberty across lower middle-income countries (LMIC) and high-income countries (HIC) have been observed in the general population [31]. Children living with HIV in LMIC have lower HAZ at ART start than those of comparable age in HIC [32], and greater growth deficits than local HIV-negative children [33]. They also experience poorer growth outcomes despite catch-up growth on ART, although catch-up growth on ART may be improved with nutritional supplementation [32]. As children and adolescents living with HIV are at increased risk of growth deficits, our results emphasize the importance, in addition to early ART initiation, of nutritional interventions to ensure full growth potential is reached throughout childhood and adolescence.

This study has several limitations. First, the inclusion criteria resulted in a select sample of ALWPHIV who started ART in childhood, survived to adolescence and remained in care. Thus, the outcomes for ALWPHIV included in the analysis likely represent a ‘best case scenario’ for outcomes of those living with HIV in the timeframe of the study. ALWPHIV were also required to have at least four height measurements in adolescence and, particularly in resource-limited settings, there may be a bias towards more frequent recording of heights in ALWPHIV who were stunted. Differences in data quality and availability across regions may also mean that those included from some regions are more representative than in other regions. We also required a height measurement after age 12 years for female individuals and after 14 years for male individuals. The differing cut-offs allowed us to increase the number of female individuals included, who on average experience earlier growth spurts than male individuals, but does mean there may be differences in the populations represented by the male and female individuals included in the analysis. In addition, adolescents represented in this analysis, who had started ART at a median age of 8 years, may be different from subsequent generations who are more likely to start ART in infancy or early childhood under ‘treat all’ approaches and have access to newer integrase inhibitor-based ART, leading to better outcomes [34]. Although low HAZ and BMIz at ART initiation were associated with pubertal growth deficits independent of age at ART start, these findings may not be generalizable to subsequent generations who will have started ART at ages much younger than in our data.

Second, WHO growth standards [17] and growth reference [18] were used to derive HAZ and BMIz. Although these references were developed to be applicable globally, they may not be the most appropriate reference for specific populations. In particular, children in Asia-Pacific had lower *z* scores than in other regions. In previous analyses conducted in paediatric HIV cohorts in Europe and Thailand in EPPICC, the associations between stunting and pubertal growth modelled using SITAR models were similar when WHO and Thai specific reference data were used [11].

Third, this study lacks comparator data from HIV-uninfected adolescents. Although we observed regional variations in growth in ALWPHIV, we were unable to assess whether differences between ALWHIC and their uninfected peers differ across regions.

Fourth, no direct measurement of puberty was available, and growth velocity was used as a proxy for timing of puberty. However, differences in timing of the pubertal growth spurt estimated from SITAR models are highly correlated with age at peak height velocity [4], which itself is correlated with (and is often used as a proxy) with timing of puberty [7]. Fifth, the analysis involved estimating SITAR parameters and using these estimates as outcomes in further analyses, which did not account for uncertainty in the SITAR estimates. Sixth, SITAR models included a spline with four degrees of freedom; models with more complex splines failed to converge overall and within some regions. Previous SITAR analyses have found better fit with splines with five or six degrees of freedom [11,23]. Finally, we lacked biological (metabolic and hormonal) data to explain in greater depth the mechanisms of growth differences according to sex.

Despite these limitations, the inclusion of ALWPHIV from a variety of settings across the globe is a key strength, giving insight into variations in growth globally, in a group for which long-term growth outcomes on ART have not been well described. With widening access to ART, the results of this study reinforce the importance of early HIV diagnosis and initiation of ART to minimize the risk of growth deficiencies in early childhood, deficiencies associated with higher risk of poorer health outcomes later in life. Longer term follow-up of adolescents living with perinatally acqiured HIV as they age into adulthood will be important to understand the full impact of pubertal growth delays on final height in adulthood and health consequences.

## Acknowledgements

The authors thank all participating networks, clinics, clinic personnel and patients who contributed data and made the Collaborative Initiative for Paediatric HIV Education and Research (CIPHER) Global Cohort Collaboration Adolescent Project possible. We also thank the CIPHER Steering Committee and CIPHER Executive Committee. The study was sponsored by the International AIDS Society-CIPHER. The full list of acknowledgments is available in the Supplement.

We would like to acknowledge George R. Seage III (1957–2021) for his contributions to the CIPHER Global Cohort Collaboration as the Principal Investigator of the Harvard CIPHER Data Center and the Pediatric HIV/AIDS Cohort Study Data and Operations Center. His leadership, collaborative spirit and contributions to understanding the long-term effects of perinatal HIV infection and antiretroviral treatment has improved care and antiretroviral drug safety guidelines for children, young adults and families all over the world. He has been sorely missed by the CIPHER community since his death.

Data availability: data are accessible in principle by applying to the Collaborative Initiative for Paediatric HIV Education and Research (CIPHER) Global Cohort Collaboration Data Centres. The CIPHER Global Cohort Collaboration is a multinetwork, multisite collaboration and this study combined data from different sites. The data do not belong to the CIPHER Global Cohort Collaboration itself; data ownership remains with the participating sites. Each site has approval from its own local Institutional Review Board to collect routine data on patients and to transfer those data anonymously to the CIPHER Global Cohort Collaboration Project University of Cape Town Research Centre (Cape Town, South Africa). For some sites and networks, Institutional Review Board approval for use of this data is restricted to the specific protocols approved to protect patient identities. Requests for access to data can be directed to the corresponding author.

∗Collaborative Initiative for Paediatric HIV Education and Research (CIPHER) Global Cohort Collaboration author contributions.

Project team: Siobhan Crichton (MRC Clinical Trials Unit at UCL, UK); Julie Jesson (CERPOP, Inserm, Université de Toulouse, Université Paul Sabatier Toulouse 3, France); Marie-Hélène Aké-Assi (University Hospital Yopougon, Abidjan, Côte d’Ivoire); Erik Belfrage (Department of Pediatrics, Karolinska Instritutet and University Hospital, Sweden); Mary-Ann Davies (School of Public Health and Family Medicine, Faculty of Health Sciences, University of Cape Town, South Africa); Jorge Pinto (School of Medicine, Federal University of Minas Gerais, Belo Horizonte, Brazil); Chloe Teasdale (CUNY Graduate School of Public Health and Health Policy, New York, NY USA); Nguyen Van Lam (National Hospital of Pediatrics, Hanoi, Vietnam); Rachel Vreeman (Icahn School of Medicine at Mount Sinai, New York, USA); Mary Paul (Baylor International Pediatric AIDS Initiative at Texas Children's Hospital, USA); Paige Williams (Harvard T. H. Chan School of Public Health, Boston, USA); Marcel Yotebieng (Division of General Internal Medicine, Department of Medicine, Albert Einstein College of Medicine, Bronx, NY, USA); Valériane Leroy^†^ (CERPOP, Inserm, Université de Toulouse, Université Paul Sabatier Toulouse 3, France); Ruth Goodall^†^ (MRC Clinical Trials Unit at UCL, UK)

† Project co-chairs, should be considered joint senior author.

Other author contributors: Elaine Abrams (ICAP at Columbia University, Mailman School of Public Health, Columbia University, New York, NY, United States of America); Russell Van Dyke (Tulane University School of Medicine, USA); Ali Judd (MRC Clinical Trials Unit at UCL, UK); Marissa Vicari (International AIDS Society, Switzerland); Intira Jeannie Collins (MRC Clinical Trials Unit at UCL, UK); Kara Wools-Kaloustian (Indiana University School of Medicine, USA); Kunjal Patel (Harvard T. H. Chan School of Public Health, USA); Amy Slogrove (Department of Paediatrics & Child Health, Faculty of Medicine & Health Sciences, Stellenbosch University, Worcester, South Africa); Kathleen M. Powis (Harvard T. H. Chan School of Public Health, USA); Mogomotsi Matshaba (Baylor College of Medicine Children's Foundation-Botswana); Lineo Thahane (Baylor College of Medicine Children's Foundation-Lesotho); Phoebe Nyasulu (Baylor College of Medicine Children's Foundation-Malawi); Bhekumusa Lukhele (Baylor College of Medicine Children's Foundation-eSwatini); Lumumba Mwita (Baylor College of Medicine Children's Foundation-Tanzania); Adeodata Kekitiinwa-Rukyalekere (Baylor College of Medicine Children's Foundation – Uganda); Tessa Goetghebuer (Hospital St Pierre, Brussels, Belgium); Claire Thorne (UCL Great Ormond Street Institute of Child Health, University College London, UK); Josiane Warszawski (Inserm U1018, Centre de recherche en Epidémiologie et Santé des Populations, France); Elena Chiappini (Meyer Chilndre's Hospital, Department of Health Science, Florence, Italy); Annemarie van Rossum (Erasmus MC University Medical Center Rotterdam-Sophia Children's Hospital, Rotterdam, The Netherlands); Magdalena Marczynska (Medical University of Warsaw, Hospital of Infectious Diseases in Warsaw, Poland); Laura Marques (Centro Hospitalar do Porto, Portugal); Filipa Prata (Hospital de Santa Maria, Lisboa, Portugal); Luminita Ene (Victor Babes Hospital, Bucharest, Romania); Liubov Okhonskaya (Republican Hospital of Infectious Diseases, St Petersburg, Russian Federation); Marisa Navarro (Hospital General Universitario ‘Gregorio Marañón’, IISGM, UCM, CIBERINFEC ISCIII, Madrid, Spain); Maria Méndez (Pediatrics Department, Hospital Universitari Germans Trias i Pujol, Universitat Autònoma de Barcelona; Badalona, Spain), Paolo Paioni (University Children's Hospital, Zurich, Switzerland); Sophie Le Coeur (Institut National d’Etude Demographique (INED), Mortality, Health and Epidemiology Unit, Paris, France, Institut de Recherche pour le Developpement (IRD), UMI-174/PHPT, Faculty of Associated Medical Sciences, Chiang Mai University, Chiang Mai, Thailand); Alla Volokha (Shupyk National Medical Academy of Postgraduate Education, Kiev); Jean William Pape (GHESKIO Center, Port-au-Prince, Haiti); Vanessa Rouzier (GHESKIO Center, Port-au-Prince, Haiti); Adias Marcelin (GHESKIO Center, Port-au-Prince, Haiti); Kulkanya Chokephaibulkit (Faculty of Medicine, Siriraj Hospital, Mahidol University, Bangkok, Thailand); Annette H. Sohn (TREAT Asia/amfAR, Bangkok, Thailand); Azar Kariminia (Kirby Institute, University of New South Wales, Sydney, Australia); Andrew Edmonds (Gillings School of Global Public Health, University of North Carolina at Chapel Hill, USA); Patricia Lelo (Pediatric Hospital Kalembe Lembe, Lingwala, Kinshasa, Democratic Republic of Congo); Francesca Akoth Odhiambo (Center for Microbiology Research, Kenya Medical Research Institute, Nairobi, Kenya); Andreas D Haas (Institute of Social and Preventive Medicine, University of Bern, Switzerland); Carolyn Bolton (Centre for Infectious Disease Research in Zambia, Lusaka, Zambia); Mariam Sylla (CHU Gabriel Toure, Bamako, Mali), Léhila Bagnan Tossa (CNHU Cotonou, Benin); Lorna Renner (Korle Bu Teaching Hospital, Accra, Ghana); Mark J. Abzug (University of Colorado School of Medicine and Children's Hospital Colorado, USA); James Oleske (Rutgers - New Jersey Medical School, USA), Murli Purswani (Bronx-Lebanon Hospital Center, USA); Elaine Chadwick (Northwestern University Feinberg School of Medicine, USA).

Authors’ contributions: S.C. conducted the analysis and wrote the article, with support from J.J. and under the supervision of V.L. and R.G. Both S.C. and R.G. contributed to the study design and protocol development. Within the project team M.Y. represents the IeDEA Central African cohort. P.W. represents the IMPAACT and PHACS cohort, C.Te. represents Optimal Models. N.V.L. represents the IeDEA Asia-Pacific cohort. M.h.Aa., V.L. and J.J. represent the IeDEA West African cohort. J.P. represents CCASAnet. E.B., R.G. and S.C. represent the EPPICC cohort. M.P. represents BIPAI. R.V. represents the IeDEA East Africa cohort. Ma.D. represents the IeDEA Southern Africa cohort.

All project team members contributed to the interpretation of the results, and subsequently revised the manuscript. All authors listed have contributed sufficiently to the conception, design, data collection, analysis, writing and/or review of the manuscript to take public responsibility for it. All author contributors have reviewed and approved the final manuscript.

Funding: this work was supported by the International AIDS Society – Collaborative Initiative for Paediatric HIV Education & Research (IAS-CIPHER, http://www.iasociety.org/CIPHER), which is made possible through funding from CIPHER Founding Sponsor ViiV Healthcare (https://www.viivhealthcare.com) and Janssen (http://www.janssen.com). The MRC Clinical Trials Unit at UCL is supported by the Medical Research Council (programme number MC_UU_00004/03).

Individual networks contributing to the CIPHER Cohort Collaboration have received the following financial support: The International Epidemiology Databases to Evaluate AIDS (IeDEA) is supported by the U.S. National Institutes of Health's National Institute of Allergy and Infectious Diseases, the *Eunice Kennedy Shriver* National Institute of Child Health and Human Development, the National Cancer Institute, the National Institute of Mental Health, the National Institute on Drug Abuse, the National Heart, Lung, and Blood Institute, the National Institute on Alcohol Abuse and Alcoholism, the National Institute of Diabetes and Digestive and Kidney Diseases, the Fogarty International Center, and the National Library of Medicine: Asia-Pacific, U01AI069907; CCASAnet, U01AI069923; Central Africa, U01AI096299; East Africa, U01AI069911; Southern Africa, U01AI069924; West Africa, U01AI069919. Overall support for the International Maternal Pediatric Adolescent AIDS Clinical Trials Network (IMPAACT) was provided by the National Institute of Allergy and Infectious Diseases (NIAID) with co-funding from the *Eunice Kennedy Shriver* National Institute of Child Health and Human Development (NICHD) and the National Institute of Mental Health (NIMH), all components of the NIH, under Award Numbers UM1AI068632 (IMPAACT LOC), UM1AI068616 (IMPAACT SDMC) and UM1AI106716 (IMPAACT LC), and by NICHD contract number HHSN275201800001I. PHACS receives funding from the US NIH (grant numbers U01 HD052102, U01 HD052104, and P01 HD103133); The Optimal Models (ICAP at Columbia University) project was supported by the President's Emergency Plan for AIDS Relief (PEPFAR) through the Centers for Disease Control and Prevention (cooperative agreement numbers: 5U62PS223540 and 5U2GPS001537); EPPICC receives funding from the PENTA Foundation (http://penta-id.org), and received support from the European Union's Horizon 2020 research and innovation programme under Grant Agreement No 825579 for the REACH study. The MRC Clinical Trials Unit at UCL is supported by the Medical Research Council (programme number MC_UU_00004/03). Individual cohorts contributing to EPPICC receive the following support: The ATHENA database is maintained by Stichting HIV Monitoring and supported by a grant from the Dutch Ministry of Health, Welfare and Sport through the Centre for Infectious Disease Control of the National Institute for Public Health and the Environment. CoRISPE-cat receives financial support from the Instituto de Salud Carlos III through the Red Temática de Investigación Cooperativa en Sida (grant numbers RED RIS RD06/0006/0035 yRD06/0006/0021). Financial support for CoRISpeS and Madrid Cohort was provided by the Instituto de Salud Carlos III through the Red Tematica de Investigacion Cooperativa en Sida (RED-RIS) project (grants number RD16/0025/0019) as part of the Plan R+D+I and cofinanced by ISCIII- Subdireccion General de Evaluación and Fondo Europeo de Desarrollo Regional (FEDER). FIS PI19/01530. The Swiss HIV Cohort Study is supported by the Swiss National Science Foundation (grant number: 177499), and by the SHCS Research Foundation. The Thai cohort study was funded by the Global Fund to fight AIDS, Tuberculosis and Malaria, Thailand (PR-A-N-008); Oxfam Great Britain, Thailand (THAA51); Ministry of Public Health, Thailand; and Institut de Recherche pour le Développement (IRD), France. Ukraine Paediatric HV Cohort is supported by the PENTA Foundation. CHIPS is funded by the NHS (London Specialised Commissioning Group) and has received additional support from Bristol-Myers Squibb, Boehringer Ingelheim, GlaxoSmithKline, Roche, Abbott and Gilead Sciences. The MRC Clinical Trials Unit at UCL is supported by the Medical Research Council (programme number MC_UU_00004/03).

No funding bodies had any role in study design, data collection and analysis, decision to publish, or preparation of the manuscript. This work is solely the responsibility of the authors and does not necessarily represent the official views of any of the institutions mentioned above.

### Conflicts of interest

M.V.'s work at CIPHER is funded through Unrestricted Educational grants received from ViiV Healthcare and Janssen to the International AIDS Society. A.So's, institution receives research funding from ViiV Healthcare. C.Th. has received grant funding from ViiV Healthcare via the Penta Foundation (to UCL). A.J. reports grants from Abbvie, Bristol Myers Squibb, Gilead, Janssen Pharmaceuticals and ViiV Healthcare through the PENTA Foundation, and from the European and Developing Countries Clinical Trials Partnership, Gilead Sciences, the International AIDS Society, NHS England, Medical Research Council and PENTA Foundation outside the submitted work. All monies were paid to her institution. I.J.C. received grants from the following companies (via her institution) in the past 3 years: ViiV, AbbVie, Gilead.

## Supplementary Material

Supplemental Digital Content
