# Supplementary material for: Global variations in pubertal growth spurts in adolescents living with perinatal HIV
Source: AIDS. 2023 May 17;37(10):1603–15. doi: 10.1097/QAD.0000000000003602 (PMC10355801; doi:10.1097/QAD.0000000000003602)
Supplement: Supplemental Digital Content [file aids-37-1603-s002.docx]

**Supplementary data**

**Supplement S1 – SITAR growth model**

Linear growth was modelled using Super Imposition by Translation And Rotation (SITAR) models ^[1]^. SITAR quantifies differences in growth via three parameters that represent average height, timing and intensity of the adolescent growth spurt (i.e. the period of rapid gains in height typically observed during adolescence). SITAR models can explain up to 99% of the variation between individuals’ growth ^[1]^ and can be summarised as:

$$y_{it}=a_{i}+h\left( \frac{t-b_{i}}{{exp}^{{-c}_{i}}} \right)$$

where the outcome *y_it_* represents the height of individual *i* at age *t* and h() is a natural cubic spline of height over age. In our analysis, a spline with 4 degrees of freedom was used (models with >4 degrees of freedom failed to converge). Parameters$a_{i}$, $b_{i}$ and $c_{i}$ are random effects representing differences between the population average and an individual’s average height, timing and intensity. Larger values of $a_{i}$ indicate taller height, on average, throughout adolescence and larger $b_{i}$ indicates later growth spurts. $c_{i}$ represents intensity and a shrinking or stretching of the age scale. Large values of $c_{i}$ indicate steeper, more rapid, growth spurts with higher growth velocity while lower values result in a shallower curve.

All height measurements (in centimetres) from age 8 (or after start of ART if later) to <19 years were included. Log transformations of age and height were considered but the untransformed data provided the best fit based on adjusted Bayesian information criterion.

Growth velocity curves were estimated by taking the first derivative of the mean height curves.

1. Cole TJ, Donaldson MD, Ben-Shlomo Y. **SITAR—a useful instrument for growth curve analysis**. *International journal of epidemiology* 2010; 39(6):1558-1566.

**Table S1:** Characteristics of females included and excluded from analysis

|  | **All**  **(n=6,732)** | **East & Southern Africa (excluding Botswana & South Africa)**  **(n=3,747)** | **Botswana & South Africa**  **(n=1,176)** | **West & Central Africa**  **(n=397)** | **Europe &**  **N America**  **(n=3747)** | **Asia-Pacific**  **(n=534)** | **Central & South America, & the Caribbean**  **(n=241)** |
| --- | --- | --- | --- | --- | --- | --- | --- |
|  |  | Median[IQR] or n(%) | | | | | |
| All |  |  |  |  |  |  |  |
| Included  Excluded | 3,230 (48%)  3,502(52%) | 1,706(46%)  2,041(54%) | 553(47%)  623(53%) | 211(53%)  186(47%) | 297(47%)  340(53%) | 342(64%)  192(36%) | 121(50%)  120(50%) |
| Year of birth |  |  |  |  |  |  |  |
| Included | 1999[1997,2000] | 1999[1998,2000] | 1998[1997,2001] | 1998[1997,1999] | 1997[1995,1999] | 1999[1998,2000] | 1999[1997,2000] |
| Excluded | 2000[1998,2001] | 2000[1999,2001] | 1999[1998,2000] | 1998[1997,1999] | 1999[1996,2001] | 2000[1998,2001] | 1998[1997,2000] |
|  |  |  |  |  |  |  |  |
| **At ART initiation** |  |  |  |  |  |  |  |
| Age (years)  Included  Excluded | 7.7[6.0,8.9]  7.4[5.5,8.8] | 8.1[6.8,9.1]  7.9[6.4,9.0] | 7.8[5.8,8.8]  7.2[5.3,8.6] | 8.0[6.6,9.0]  7.6[6.2,8.9] | 5.3[2.4,7.8]  3.7[0.8,6.8] | 6.5[5.0,7.9]  6.1[4.3,7.9] | 4.1[1.7,7.1]  6.1[3.8,8.1] |
| PI regimen  Included  Excluded | 266(8%)  363(10%) | 23(1%)  16(1%) | 29(5%)  53(9%) | 17(8%)  60(32%) | 143(48%)  203(60%) | 4(1%)  11(6%) | 50(41%)  20(17%) |
| HAZ  Included  Excluded | -2.0[-2.9,-1.1]  -2.0[-3.0,-1.1] | -2.1[-3.0,-1.2]  -2.0[-2.9,-1.0] | -2.0[-2.7,-1.2]  -2.1[-3.1,-1.3] | -1.6[-2.6,-0.6]  -2.0[-2.7,-1.3] | -1.0[-1.7,0.0]  -0.2[-1.4,0.4] | -2.3[-3.3,-1.5]  -2.2[-3.4,-1.4] | -1.8[-2.7,-0.9]  -1.8[-2.1,-1.0] |
| BMIz  Included  Excluded | -0.6[-1.5,0.1]  -0.6[-1.4,0.2] | -0.7[-1.5,0.3]  -0.7[-1.6,0.1] | -0.5[-1.3,0.2]  -0.3[-1.0,0.5] | -1.3[-2.2,-0.4]  -1.1[-2.1,-0.3] | 0.1[-0.6,0.9]  0.4[-0.7,1.3] | -0.9[-1.7,-0.1]  -0.8[-2.3,0.2] | -0.4[-1.2,0.6]  -0.5[-0.9,-0.2] |
| In total, 6732 females were born ≥ 12 years prior to end of follow-up and potentially eligible for the analysis. 3230 had complete data at ART initiation and at least 4 heights recorded during follow-up and were included in analysis. In this table, characteristics of the 3230 included are compared to 3502 who were excluded due to missing data. HAZ and BMI at ART initiation was available for 1519 and 1476 females, respectively, excluded from the models. Abbreviations: ART Antiretroviral therapy; BMIz Body Mass Index -for-age z-score; HAZ Height-for-age z-score; IQR Interquartile range; PI boosted protease inhibitor | | | | | | | |

**Table S2** Characteristics of males included and excluded from analysis

|  | **All**  **(n=3,879)** | **East & Southern Africa (excluding Botswana & South Africa)**  **(n=1,975)** | **Botswana & South Africa**  **(n=765)** | **West & Central Africa**  **(n=208)** | **Europe &**  **N America**  **(n=477)** | **Asia & Pacific**  **(n=310)** | **Central & South America, & the Caribbean**  **(n=144)** |
| --- | --- | --- | --- | --- | --- | --- | --- |
|  |  | Median[IQR] or n(%) | | | | | |
| All |  |  |  |  |  |  |  |
| Included  Excluded | 1,493(38%)  2,386(62%) | 704(36%)  1,271(64%) | 263(34%)  502(66%) | 100(48%)  108(52%) | 208(44%)  269(56%) | 160(52%)  150 (48%) | 58(40%)  86(60%) |
| Year of birth |  |  |  |  |  |  |  |
| Included | 1998[1996,1999] | 1998[1997,1999] | 1997[1996,1999] | 1997[1996,1998] | 1996[1993,1997] | 1998[1997,1999] | 1998[1996,1998] |
| Excluded | 1998[1997,1999] | 1999[1998,1999] | 1998[1997,1999] | 1997[1995,1997] | 1997[1995,1999] | 1999[1997,2000] | 1998[1997,1999] |
|  | | | | | | | |
| **At ART initiation** | | | | | | | |
| Age (years)  Included  Excluded | 8.0[6.6,9.1]  7.9[6.3,9.0] | 8.4[7.4,9.3]  8.4[7.2,9.2] | 8.1[6.4,9.1]  7.3[6.0,8.7] | 8.6[7.6,9.4]  8.4[7.4,9.1] | 5.7[3.2,8.0]  4.4[1.3,7.0] | 7.1[5.9,8.2]  7.0[5.6,8.2] | 5.3[3.2,7.4] 5.7[4.0,7.5] |
| PI regimen  Included  Excluded | 182(12%)  270(11%) | 27(4%)  20(2%) | 13(5%)  38(8%) | 14(14%)  38(35%) | 108(52%)  151(56%) | 1(1%)  5(3%) | 19(33%)  18(21%) |
| HAZ  Included  Excluded | -1.8[-2.8,-1.0]  -2.0[-3.0,-1.1] | -2.1[-2.9,-1.2]  -2.1[-3.0,-1.1] | -1.8[-2.7,-1.1]  -2.1[-3.0,-1.3] | -1.6[-2.7,-0.8]  -2.2[-2.9,-1.2] | -0.8[-1.4,0.1]  -0.8[-1.7,0.1] | -2.5[-3.3,-1.6]  -2.3[-3.2,-1.5] | -1.8[-2.7,-1.2]  -1.7[-2.7,-1.1] |
| BMIz  Included  Excluded | -0.7[-1.6,0.2]  -0.7[-1.6,0.1] | -0.8[-1.8,0.0]  -0.8[-1.8,0.0] | -0.7[-1.4,0.2]  -0.4[-1.1,0.3] | -1.5[-2.2,-0.7]  -1.4[-2.6,-0.7] | 0.3[-0.5,1.1]  0.2[-0.5,0.7] | -1.0[-1.9,-0.1]  -1.0[-2.4,-0.4] | -0.3[-1.5,0.3]  -0.3[-1.3,0.3] |
| In total 3879 males were born ≥ 14 years prior to end of follow-up and potentially eligible for the analysis. 1493 had complete data at ART initiation and at least 4 heights recorded during follow-up and were included in analysis. In this table characteristics of the 1493 included are compared to 2386 who were excluded due to missing data. HAZ and BMI at ART initiation was available for 1218 and 1183 males, respectively, excluded from the models. Abbreviations: ART Antiretroviral therapy; BMIz Body Mass Index -for-age z-score; HAZ Height-for-age z-score; IQR Interquartile range; PI boosted protease inhibitor | | | | | | | |

**Table S3:** Availability of height data

|  | Number of height measurements | | | | Age at latest height measurement | | | |
| --- | --- | --- | --- | --- | --- | --- | --- | --- |
|  | Median | IQR | Min | Max | Median | IQR | Min | Max |
| **Females** |  |  |  |  |  |  |  |  |
| All | 27 | [18,38] | 4 | 108 | 13.9 | [12.8,15.2] | 12.0 | 19.0 |
| Region |  |  |  |  |  |  |  |  |
| East & Southern Africa | 29 | [19,41] | 4 | 108 | 13.6 | [12.7,14.8] | 12.0 | 19.0 |
| Botswana & South Africa | 27 | [22,34] | 4 | 79 | 14.0 | [12.9,15.7] | 12.0 | 19.0 |
| West & Central Africa | 20 | [12,46] | 4 | 102 | 13.6 | [12.7,14.7] | 12.0 | 18.7 |
| Europe & North America | 19 | [12,27] | 4 | 66 | 15.4 | [13.8,16.8] | 12.0 | 19.0 |
| Asia-Pacific | 29 | [19,41] | 4 | 93 | 14.2 | [13.0,15.2] | 12.0 | 19.0 |
| Central & South America, & the Caribbean | 24 | [18,36] | 8 | 69 | 14.3 | [13.1,16.6] | 12.1 | 19.0 |
|  |  |  |  |  |  |  |  |  |
| **Males** |  |  |  |  |  |  |  |  |
| All | 33 | [23,44] | 4 | 116 | 15.3 | [14.6,16.4] | 14.0 | 19.0 |
| Region |  |  |  |  |  |  |  |  |
| East & Southern Africa | 37 | [24,50] | 4 | 116 | 15.1 | [14.5,16.1] | 14.0 | 19.0 |
| Botswana & South Africa | 34 | [29,39] | 5 | 77 | 15.5 | [14.7,17.2] | 14.0 | 19.0 |
| West & Central Africa | 26 | [16,64] | 5 | 97 | 15.0 | [14.5,15.6] | 14.0 | 18.5 |
| Europe & North America | 23 | [16,30] | 4 | 52 | 16.4 | [15.3,17.6] | 14.0 | 19.0 |
| Asia-Pacific | 36 | [26,48] | 11 | 106 | 15.1 | [14.5,16.0] | 14.0 | 19.0 |
| Central & South America, & the Caribbean | 30 | [25,37] | 14 | 70 | 15.4 | [14.8,16.6] | 14.0 | 18.9 |

.


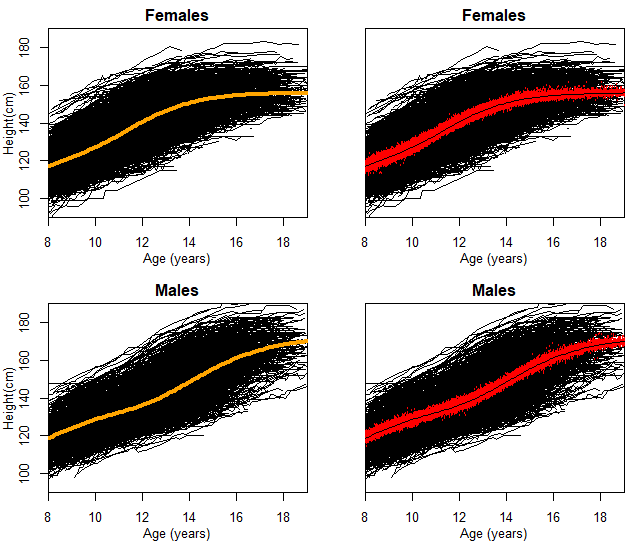


On the left, individual growth trajectories are shown along with a fitted spline. In females, the residual standard deviation (RSD) around this curve was 7.9 cm, and in males it was 8.4 cm. On the right SITAR adjusted height measurements are shown in red. In females, the RSD after adjustment was 0.9 cm, and it was 1.0 cm in males, corresponding to a reduction in variation of 97.9% and 98.0%, respectively.

**Figure S1:** Individual height trajectory and SITAR adjusted height measurements

**Table S4:** Regional variations in average height, timing, and intensity of the pubertal growth

|  | Females | | | |  | Males | | | |
| --- | --- | --- | --- | --- | --- | --- | --- | --- | --- |
|  | Coef. | Lower 95% CI | Upper 95% CI | p-value |  | Coef. | Lower 95% CI | Upper 95% CI | p-value |
| **a - average height** |  |  |  |  |  |  |  |  |  |
| East & Southern Africa | ref |  |  |  |  | ref |  |  |  |
| Botswana & South Africa | 3.602 | 2.752 | 4.452 | <0.001 |  | 1.557 | 0.103 | 3.011 | 0.036 |
| West & Central Africa | 4.979 | 3.712 | 6.246 | <0.001 |  | 3.671 | 1.521 | 5.820 | 0.001 |
| Europe & North America | 8.299 | 7.207 | 9.390 | <0.001 |  | 9.345 | 7.757 | 10.933 | <0.001 |
| Asia-Pacific | 0.395 | -0.633 | 1.424 | 0.451 |  | -1.214 | -2.976 | 0.547 | 0.177 |
| Central & South America, & the Caribbean | 4.284 | 2.651 | 5.918 | <0.001 |  | 0.643 | -2.105 | 3.391 | 0.647 |
| **b –timing of growth spurt** |  |  |  |  |  |  |  |  |  |
| East & Southern Africa | ref |  |  |  |  | ref |  |  |  |
| Botswana & South Africa | 0.434 | 0.306 | 0.563 | <0.001 |  | -0.003 | -0.267 | 0.261 | 0.980 |
| West & Central Africa | 0.221 | 0.029 | 0.413 | 0.024 |  | 0.038 | -0.353 | 0.428 | 0.850 |
| Europe & North America | -0.370 | -0.536 | -0.205 | <0.001 |  | -0.494 | -0.783 | -0.206 | 0.001 |
| Asia-Pacific | -0.009 | -0.165 | 0.147 | 0.912 |  | -0.337 | -0.657 | -0.017 | 0.039 |
| Central & South America, & the Caribbean | -0.310 | -0.558 | -0.063 | 0.014 |  | -0.946 | -1.445 | -0.447 | <0.001 |
| **c –Intensity of growth spurt** |  |  |  |  |  |  |  |  |  |
| East & Southern Africa | ref |  |  |  |  | ref |  |  |  |
| Botswana & South Africa | -0.002 | -0.023 | 0.018 | 0.825 |  | 0.033 | 0.007 | 0.059 | 0.013 |
| West & Central Africa | 0.037 | 0.007 | 0.068 | 0.016 |  | 0.027 | -0.012 | 0.065 | 0.178 |
| Europe & North America | 0.095 | 0.069 | 0.121 | <0.001 |  | 0.111 | 0.082 | 0.139 | <0.001 |
| Asia-Pacific | 0.060 | 0.035 | 0.084 | <0.001 |  | 0.139 | 0.107 | 0.170 | <0.001 |
| Central & South America, & the Caribbean | 0.076 | 0.037 | 0.116 | <0.001 |  | 0.094 | 0.044 | 0.143 | <0.001 |
|  | | | | | | | | | |

**Table S5:** Multivariable analysis of association between characteristics at ART initiation and timing of the pubertal growth in females

|  | Coef. | Standard error | t-value | p-value | Lower 95% CI | Upper 95% CI |
| --- | --- | --- | --- | --- | --- | --- |
| Region |  |  |  |  |  |  |
| East & Southern Africa | ref |  |  |  |  |  |
| Botswana & South Africa | -1.102 | 0.375 | -2.94 | 0.003 | -1.838 | -0.366 |
| West & Central Africa | -0.869 | 0.731 | -1.19 | 0.235 | -2.303 | 0.565 |
| Europe & North America | -1.640 | 0.323 | -5.08 | <0.001 | -2.273 | -1.007 |
| Asia-Pacific | -1.754 | 0.414 | -4.24 | <0.001 | -2.565 | -0.943 |
| Central & South America, & the Caribbean | -2.160 | 0.473 | -4.57 | <0.001 | -3.087 | -1.233 |
|  |  |  |  |  |  |  |
|  |  |  |  |  |  |  |
| Initiated ART on PI based regimen | 0.047 | 0.100 | 0.47 | 0.639 | -0.149 | 0.243 |
|  |  |  |  |  |  |  |
|  |  |  |  |  |  |  |
| HAZ at ART initiation (FP comp 1) | -0.067 | 0.009 | -7.76 | <0.001 | -0.084 | -0.050 |
| HAZ at ART initiation (FP comp 2) | -0.011 | 0.001 | -7.57 | <0.001 | -0.014 | -0.008 |
|  |  |  |  |  |  |  |
|  |  |  |  |  |  |  |
| Age at ART initiation (FP comp 1) | -0.015 | 0.003 | -4.81 | <0.001 | -0.021 | -0.009 |
| Age at ART initiation (FP comp 2) | 0.006 | 0.001 | 4.82 | <0.001 | 0.004 | 0.009 |
|  |  |  |  |  |  |  |
|  |  |  |  |  |  |  |
| BMIz at ART initiation | 0.009 | 0.047 | 0.20 | 0.841 | -0.083 | 0.102 |
|  |  |  |  |  |  |  |
| Year of birth (FP comp 1) | -0.291 | 0.041 | -7.10 | <0.001 | -0.372 | -0.211 |
|  |  |  |  |  |  |  |
|  |  |  |  |  |  |  |
| Region X Age at ART initiation |  |  |  |  |  |  |
| Botswana & South Africa X Age FP Comp 1 | 0.016 | 0.005 | 3.32 | 0.001 | 0.007 | 0.026 |
| West & Central Africa X Age FP Comp 1 | 0.016 | 0.009 | 1.69 | 0.091 | -0.003 | 0.034 |
| Europe & North America X Age FP Comp 1 | 0.012 | 0.005 | 2.33 | 0.020 | 0.002 | 0.022 |
| Asia-Pacific X Age FP Comp 1 | 0.015 | 0.005 | 2.80 | 0.005 | 0.005 | 0.026 |
| Central & South America, & the Caribbean X Age FP Comp 1 | 0.019 | 0.008 | 2.49 | 0.013 | 0.004 | 0.034 |
| Botswana & South Africa X Age FP Comp 2 | -0.007 | 0.002 | -3.30 | 0.001 | -0.011 | -0.003 |
| West & Central Africa X Age FP Comp 2 | -0.007 | 0.004 | -1.74 | 0.082 | -0.014 | 0.001 |
| Europe & North America X Age FP Comp 2 | -0.005 | 0.002 | -2.17 | 0.030 | -0.009 | 0.000 |
| Asia-Pacific X Age FP Comp 2 | -0.006 | 0.002 | -2.64 | 0.008 | -0.011 | -0.002 |
| Central, & South America, & the Caribbean X Age FP Comp 2 | -0.008 | 0.003 | -2.25 | 0.024 | -0.014 | -0.001 |
|  |  |  |  |  |  |  |
|  | |  |  |  |  |  |
| BMIz at ART initiation X Age at ART initiation | |  |  |  |  |  |
| BMIz X Age FP Comp 1 | -0.001 | 0.001 | -0.52 | 0.600 | -0.002 | 0.001 |
| BMIz X Age FP Comp 2 | 0.000 | 0.000 | 0.37 | 0.708 | -0.001 | 0.001 |
|  |  |  |  |  |  |  |
|  |  |  |  |  |  |  |
| Region X Year of birth |  |  |  |  |  |  |
| Botswana & South Africa | 0.282 | 0.073 | 3.88 | <0.001 | 0.139 | 0.425 |
| West & Central Africa | 0.128 | 0.157 | 0.81 | 0.416 | -0.181 | 0.437 |
| Europe & North America | 0.261 | 0.094 | 2.79 | 0.005 | 0.077 | 0.445 |
| Asia-Pacific | 0.316 | 0.101 | 3.12 | 0.002 | 0.118 | 0.514 |
| Central & South America, & the Caribbean | 0.328 | 0.139 | 2.36 | 0.019 | 0.055 | 0.600 |
| A multivariable linear regression model was used to analyse factors associated with a parameter representing timing of the pubertal growth spurt estimated using SITAR. Coefficients represent differences in timing (in years) with lower values indicating earlier growth spurts and higher values indicating later growth spurts. Age and HAZ (transformed as -HAZ/SD(HAZ), where SD(HAZ)=1.5) at ART initiation were modelled using second order fractional polynomials (FP(3 3) and FP(-1 -1), respectively) and year of birth as a first degree fractional polynomial (FP(3)). Model includes interactions between region and age at ART initiation (LRT, p=0.0002), BMIz and age at ART initiation (p=0.0250) and region and year of birth (p<0.001). Abbreviations: ART Antiretroviral therapy; BMIz Body Mass Index -for-age z-score; HAZ Height-for-age z-score; PI boosted protease inhibitor | | | | | | |

**Table S6:** Multivariable analysis of association between characteristics at ART initiation and intensity of the pubertal growth in females

|  | Coef. | Standard error | t-value | p-value | Lower 95% CI | Upper 95% CI |
| --- | --- | --- | --- | --- | --- | --- |
| Region |  |  |  |  |  |  |
| East & Southern Africa | ref |  |  |  |  |  |
| Botswana & South Africa | -0.224 | 0.062 | -3.61 | 0.000 | -0.346 | -0.102 |
| West & Central Africa | -0.151 | 0.104 | -1.45 | 0.146 | -0.355 | 0.053 |
| Europe & North America | -0.104 | 0.059 | -1.76 | 0.078 | -0.219 | 0.012 |
| Asia-Pacific | 0.136 | 0.081 | 1.68 | 0.093 | -0.023 | 0.296 |
| Central & South America, & the Caribbean | -0.258 | 0.096 | -2.68 | 0.007 | -0.447 | -0.069 |
|  |  |  |  |  |  |  |
|  |  |  |  |  |  |  |
| Initiated ART on PI based regimen | -0.014 | 0.016 | -0.90 | 0.369 | -0.045 | 0.017 |
|  |  |  |  |  |  |  |
|  |  |  |  |  |  |  |
|  |  |  |  |  |  |  |
| HAZ at ART initiation | -0.003 | 0.003 | -1.00 | 0.319 | -0.010 | 0.003 |
|  |  |  |  |  |  |  |
|  |  |  |  |  |  |  |
| Age at ART initiation | 0.008 | 0.002 | 3.73 | 0.000 | 0.004 | 0.012 |
|  |  |  |  |  |  |  |
|  |  |  |  |  |  |  |
| BMIz at ART initiation (FP comp 1) | 0.093 | 0.026 | 3.61 | 0.000 | 0.042 | 0.143 |
| BMIz at ART initiation (FP comp 2) | -0.038 | 0.011 | -3.53 | 0.000 | -0.059 | -0.017 |
|  |  |  |  |  |  |  |
|  |  |  |  |  |  |  |
| Year of birth | -0.015 | 0.003 | -4.82 | 0.000 | -0.021 | -0.009 |
|  |  |  |  |  |  |  |
|  |  |  |  |  |  |  |
| Region X HAZ at ART initiation |  |  |  |  |  |  |
| Botswana & South Africa | -0.003 | 0.008 | -0.42 | 0.673 | -0.019 | 0.013 |
| West & Central Africa | 0.020 | 0.010 | 1.98 | 0.048 | 0.000 | 0.041 |
| Europe & North America | 0.006 | 0.009 | 0.64 | 0.521 | -0.012 | 0.025 |
| Asia-Pacific | 0.025 | 0.009 | 2.81 | 0.005 | 0.008 | 0.043 |
| Central & South America, & the Caribbean | -0.015 | 0.015 | -0.98 | 0.327 | -0.045 | 0.015 |
|  |  |  |  |  |  |  |
|  |  |  |  |  |  |  |
| Region X year of birth |  |  |  |  |  |  |
| Botswana & South Africa | 0.018 | 0.005 | 3.73 | 0.000 | 0.009 | 0.028 |
| West & Central Africa | 0.018 | 0.009 | 2.00 | 0.046 | 0.000 | 0.036 |
| Europe & North America | 0.021 | 0.005 | 4.01 | 0.000 | 0.011 | 0.031 |
| Asia-Pacific | -0.001 | 0.007 | -0.17 | 0.865 | -0.014 | 0.012 |
| Central & South America, & the Caribbean | 0.029 | 0.008 | 3.60 | 0.000 | 0.013 | 0.045 |
| A multivariable linear regression model was used to analyse factors associated with a parameter representing the intensity of the pubertal growth spurt estimated using SITAR. Lower values indicating lower intensity growth spurts and higher values indicating and more intense and rapid growth spurts. BMIz (transformed as -BMIz/SD(BMIz), where SD(BMIz)=1.5) at ART initiation was modelled using a second order fractional polynomials (FP(2 3)). Model includes interactions between region and HAZ at ART initiation (LRT, p<0.001) and region and year of birth (p=0.0187). Abbreviations: ART Antiretroviral therapy; BMIz Body Mass Index-for-age z-score; HAZ Height-for-age z-score; PI boosted protease inhibitor | | | | | | |

**Table S7:** Multivariable analysis of association between characteristics at ART initiation and timing of the pubertal growth in males

|  | Coef. | Standard error | t-value | p-value | Lower 95% CI | Upper 95% CI |
| --- | --- | --- | --- | --- | --- | --- |
| Region |  |  |  |  |  |  |
| East & Southern Africa | ref |  |  |  |  |  |
| Botswana & South Africa | -0.509 | 0.846 | -0.60 | 0.548 | -2.169 | 1.151 |
| West & Central Africa | -4.923 | 1.512 | -3.26 | 0.001 | -7.889 | -1.958 |
| Europe & North America | -2.112 | 0.734 | -2.88 | 0.004 | -3.551 | -0.672 |
| Asia-Pacific | -3.135 | 1.019 | -3.08 | 0.002 | -5.135 | -1.135 |
| Central & South America, & the Caribbean | -3.485 | 1.664 | -2.09 | 0.036 | -6.750 | -0.220 |
|  |  |  |  |  |  |  |
| Initiated ART on PI based regimen | -0.002 | 0.176 | -0.01 | 0.992 | -0.347 | 0.343 |
|  |  |  |  |  |  |  |
| HAZ at ART initiation (FP comp 1) | -0.014 | 0.060 | -0.23 | 0.817 | -0.133 | 0.105 |
| HAZ at ART initiation (FP comp 2) | -0.125 | 0.417 | -0.30 | 0.764 | -0.944 | 0.693 |
|  |  |  |  |  |  |  |
| Age at ART initiation (FP comp 1) | 0.035 | 0.011 | 3.04 | 0.002 | 0.012 | 0.057 |
| Age at ART initiation (FP comp 2) | -0.015 | 0.005 | -3.18 | 0.002 | -0.025 | -0.006 |
|  |  |  |  |  |  |  |
| BMIz at ART initiation | 0.000 | 0.033 | -0.01 | 0.992 | -0.066 | 0.065 |
|  |  |  |  |  |  |  |
| Year of birth | -0.155 | 0.057 | -2.70 | 0.007 | -0.267 | -0.042 |
|  |  |  |  |  |  |  |
| Region X year of birth |  |  |  |  |  |  |
| Botswana & South Africa | 0.047 | 0.079 | 0.59 | 0.553 | -0.108 | 0.201 |
| West & Central Africa | 0.502 | 0.151 | 3.31 | 0.001 | 0.204 | 0.799 |
| Europe & North America | 0.198 | 0.073 | 2.73 | 0.006 | 0.056 | 0.341 |
| Asia-Pacific | 0.244 | 0.095 | 2.57 | 0.010 | 0.058 | 0.431 |
| Central & South America & the Caribbean | 0.247 | 0.159 | 1.55 | 0.121 | -0.065 | 0.559 |
|  |  |  |  |  |  |  |
| HAZ at ART initiation X Age at ART initiation | |  |  |  |  |  |
| HAZ FP Comp 1 X Age FP Comp 1 | 0.002 | 0.001 | 1.79 | 0.073 | 0.000 | 0.004 |
| HAZ FP Comp 1 X Age FP Comp 2 | -0.001 | 0.000 | -1.87 | 0.062 | -0.002 | 0.000 |
| HAZ FP Comp 2 X Age FP Comp 1 | -0.017 | 0.008 | -2.18 | 0.029 | -0.032 | -0.002 |
| HAZ FP Comp 2 X Age FP Comp 2 | 0.007 | 0.003 | 2.28 | 0.023 | 0.001 | 0.014 |
| A multivariable linear regression model was used to analyse factors associated with a parameter representing timing of the pubertal growth spurt estimated using SITAR. Coefficients represent differences in timing (in years) with lower values indicating earlier growth spurts and higher values indicating later growth spurts. Age and HAZ (transformed as -HAZ/SD(HAZ) at ART initiation were modelled using second order fractional polynomials (FP(3 3) and FP(-1 -0.5), respectively). Model includes interactions between region and year of birth (LRT, p=0.0014) and HAZ and age at ART initiation (p=0.0387). Abbreviations: ART Antiretroviral therapy; BMIz Body Mass Index-for-age z-score; HAZ Height-for-age z-score; PI boosted protease inhibitor | | | | | | |

**Table S8:** Multivariable analysis of association between characteristics at ART initiation and intensity of the pubertal growth in males

|  | Coef. | Standard error | t-value | p-value | Lower 95% CI | Upper 95% CI |
| --- | --- | --- | --- | --- | --- | --- |
| Region |  |  |  |  |  |  |
| East & Southern Africa | ref |  |  |  |  |  |
| Botswana & South Africa | 0.028 | 0.014 | 2.05 | 0.041 | 0.001 | 0.055 |
| West & Central Africa | 0.021 | 0.020 | 1.03 | 0.305 | -0.019 | 0.060 |
| Europe & North America | 0.090 | 0.022 | 4.00 | <0.001 | 0.046 | 0.134 |
| Asia-Pacific | 0.134 | 0.017 | 7.92 | <0.001 | 0.101 | 0.168 |
| Central & South America, & the Caribbean | 0.083 | 0.028 | 2.98 | 0.003 | 0.028 | 0.137 |
|  |  |  |  |  |  |  |
| Initiated ART on PI based regimen | -0.006 | 0.018 | -0.33 | 0.745 | -0.041 | 0.029 |
|  |  |  |  |  |  |  |
| HAZ at ART initiation | 0.007 | 0.004 | 2.00 | 0.046 | 0.000 | 0.015 |
|  |  |  |  |  |  |  |
| Age at ART initiation | -0.001 | 0.003 | -0.28 | 0.782 | -0.008 | 0.006 |
|  |  |  |  |  |  |  |
| BMIz at ART initiation | -0.025 | 0.012 | -2.10 | 0.036 | -0.048 | -0.002 |
|  |  |  |  |  |  |  |
| Year of birth | -0.004 | 0.003 | -1.21 | 0.227 | -0.010 | 0.002 |
|  |  |  |  |  |  |  |
| BMIz at ART initiation X Age at ART initiation | 0.003 | 0.002 | 2.19 | 0.028 | 0.000 | 0.006 |
| A multivariable linear regression model was used to analyse factors associated with a parameter representing the intensity of the pubertal growth spurt estimated using SITAR. Lower values indicating less intense growth spurts and higher values indicating more intense and rapid growth spurts. Model includes an interaction between BMIz and age at ART initiation (LRT, p=0.0277). Abbreviations: ART Antiretroviral therapy; BMIz Body Mass Index-for-age z-score; HAZ Height-for-age z-score; PI boosted protease inhibitor | | | | | | |

On the y-axes, 0 represents the average time (A-C) and average intensity (D-F) of the growth spurt across all females in the analysis. Lower values represent earlier/less intense growth spurts and higher values later/more intense growth spurts. For example, A demonstrates that increasing age at ART is associated with later growth spurts, though there is some variation by region. D shows that increasing age at ART initiation is associated with more intense growth spurts. Lines represent differences in timing/intensity across regions for adolescents born in 1999, who initiated an NNRTI regimen, with a BMIz of -1 (in panels A, B, D, E), HAZ of -1 (A, C, D, F) at age 8 years (B, E, F). Lines are restricted to the 10^th^-90^th^ percentile of age, HAZ and BMIz observed in each region. The interaction between HAZ and age for timing (panel E) is illustrated for adolescents in East and Southern Africa and Europe and North America. Abbreviations: ART Antiretroviral therapy; BMIz Body Mass Index-for-age z-score; HAZ Height-for-age z-score; NNRTI Non-nucleoside reverse transcriptase inhibitor

**Figure S2: Female pubertal growth:** Multivariable associations between region, age at ART initiation, HAZ and BMIz at age 10 years and the timing and intensity of the pubertal growth spurt in females in the CIPHER global cohort collaboration, 1994-2015.

On the y-axes, 0 represents the average time (A-C) and average intensity (D-F) of the growth spurt across all males in the analysis. Lower values represent earlier/less intense growth spurts and higher values later/more intense growth spurts. For example, A demonstrates that increasing age at ART is associated with later growth spurts up to around age 7, and earlier growth spurts after age 7 years. D shows that increasing age at ART initiation is associated with more intense growth spurts. Lines represent differences in timing/intensity across regions for males born in 1998, who initiated an NNRTI regimen, with a BMIz of -1 (in panels A, B, D, E), HAZ of -1 (A, C, D, F) at age 8 years (B, C, E). Lines are restricted to the 10^th^-90^th^ percentile of age, HAZ and BMIz observed in each region. Abbreviations: ART Antiretroviral therapy; BMIz Body Mass Index-for-age z-score; HAZ Height-for-age z-score; NNRTI Non-nucleoside reverse transcriptase inhibitor

**Figure S3: Male pubertal growth:** Multivariable associations between region, age at ART initiation, HAZ and BMIz at age 10 years and the timing and intensity of the pubertal growth spurt in males in the CIPHER global cohort collaboration, 1994-2015.

**Table S9:** Multivariable analysis of association between characteristics at age 10 years and timing of the pubertal growth in females

|  | Coef. | Standard error | t-value | p-value | Lower 95% CI | Upper 95% CI |
| --- | --- | --- | --- | --- | --- | --- |
| Region |  |  |  |  |  |  |
| East & Southern Africa | ref |  |  |  |  |  |
| Botswana & South Africa | -2.104 | 0.639 | -3.29 | 0.001 | -3.357 | -0.851 |
| West & Central Africa | 0.406 | 1.094 | 0.37 | 0.710 | -1.738 | 2.550 |
| Europe & North America | -0.017 | 0.560 | -0.03 | 0.975 | -1.116 | 1.081 |
| Asia-Pacific | -1.397 | 0.773 | -1.81 | 0.071 | -2.913 | 0.119 |
| Central & South America, & the Caribbean | -1.811 | 0.953 | -1.9 | 0.058 | -3.679 | 0.058 |
|  |  |  |  |  |  |  |
|  |  |  |  |  |  |  |
| Initiated ART on PI based regimen | 0.047 | 0.100 | 0.47 | 0.639 | -0.149 | 0.243 |
|  |  |  |  |  |  |  |
|  |  |  |  |  |  |  |
| HAZ at age 10 years (FP comp 1) | 0.018 | 0.002 | 10 | <0.001 | 0.015 | 0.022 |
| HAZ at age 10 years (FP comp 2) | -0.467 | 0.032 | -14.65 | <0.001 | -0.529 | -0.404 |
|  |  |  |  |  |  |  |
| Age at ART initiation (FP comp 1) | -0.016 | 0.003 | -5.38 | <0.001 | -0.021 | -0.010 |
| Age at ART initiation (FP comp 2) | 0.007 | 0.001 | 5.37 | <0.001 | 0.004 | 0.009 |
|  |  |  |  |  |  |  |
| BMIz at age 10 years (FP comp 1) | 0.004 | 0.002 | 2.41 | 0.016 | 0.001 | 0.008 |
| BMIz at age 10 years (FP comp 2) | -0.155 | 0.027 | -5.69 | <0.001 | -0.208 | -0.102 |
|  |  |  |  |  |  |  |
| Year of birth (FP comp 1) | 0.384 | 0.328 | 1.17 | 0.241 | -0.259 | 1.027 |
| Year of birth (FP comp 2) | -1.105 | 0.533 | -2.07 | 0.038 | -2.150 | -0.060 |
|  |  |  |  |  |  |  |
|  |  |  |  |  |  |  |
| Region X Year of birth |  |  |  |  |  |  |
| Botswana & South Africa X year FP comp 1 | 1.318 | 0.487 | 2.71 | 0.007 | 0.363 | 2.273 |
| West & Central Africa X year FP comp 1 | -1.065 | 0.884 | -1.2 | 0.228 | -2.799 | 0.668 |
| Europe & North America X year FP comp 1 | -0.874 | 0.453 | -1.93 | 0.054 | -1.764 | 0.015 |
| Asia-Pacific X year FP comp 1 | 0.001 | 0.631 | 0 | 0.998 | -1.236 | 1.239 |
| Central & South America, & the Caribbean X year FP comp 1 | 0.388 | 0.765 | 0.51 | 0.612 | -1.112 | 1.887 |
| Botswana & South Africa X year FP comp 2 | -1.629 | 0.782 | -2.08 | 0.037 | -3.162 | -0.097 |
| West & Central Africa X year FP comp 2 | 2.028 | 1.657 | 1.22 | 0.221 | -1.221 | 5.277 |
| Europe & North America X year FP comp 2 | 2.184 | 0.832 | 2.62 | 0.009 | 0.552 | 3.816 |
| Asia-Pacific X year FP comp 2 | 0.451 | 1.065 | 0.42 | 0.672 | -1.636 | 2.538 |
| Central & South America, & the Caribbean X year FP comp 2 | -0.159 | 1.290 | -0.12 | 0.902 | -2.688 | 2.369 |
|  |  |  |  |  |  |  |
|  |  |  |  |  |  |  |
| Region X Age at ART initiation |  |  |  |  |  |  |
| Botswana & South Africa X Age FP Comp 1 | 0.011 | 0.005 | 2.29 | 0.022 | 0.002 | 0.021 |
| West & Central Africa X Age FP Comp 1 | 0.018 | 0.010 | 1.82 | 0.069 | -0.001 | 0.037 |
| Europe & North America X Age FP Comp 1 | 0.012 | 0.005 | 2.36 | 0.018 | 0.002 | 0.022 |
| Asia-Pacific X Age FP Comp 1 | 0.014 | 0.005 | 2.62 | 0.009 | 0.004 | 0.025 |
| Central & South America, & the Caribbean X Age FP Comp 1 | 0.016 | 0.008 | 2.06 | 0.040 | 0.001 | 0.030 |
| Botswana & South Africa X Age FP Comp 2 | -0.005 | 0.002 | -2.2 | 0.028 | -0.009 | 0.000 |
| West & Central Africa X Age FP Comp 2 | -0.008 | 0.004 | -1.86 | 0.063 | -0.015 | 0.000 |
| Europe & North America X Age FP Comp 2 | -0.005 | 0.002 | -2.27 | 0.023 | -0.009 | -0.001 |
| Asia-Pacific X Age FP Comp 2 | -0.006 | 0.002 | -2.43 | 0.015 | -0.010 | -0.001 |
| Central, & South America, & the Caribbean X Age FP Comp 2 | -0.006 | 0.003 | -1.83 | 0.068 | -0.013 | 0.000 |
| A multivariable linear regression model was used to analyse factors associated with a parameter representing timing of the pubertal growth spurt estimated using SITAR. Coefficients represent differences in timing (in years) with lower values indicating earlier growth spurts and higher values indicating later growth spurts. Age, HAZ (transformed as -HAZ/SD(HAZ), where SD(HAZ)=1.3) at age 10 years, and BMIz (transformed as -BMIz/SD(BMIz), where SD(BMIz)=1.1) at age 10 years and year of both were modelled using second order fractional polynomials (FP(3 3) , FP(-1 -1), FP(-1 -0.5) and FP (3 3) respectively). Model includes interactions between region and age at ART initiation (LRT, p=0.0029), and region and year of birth (p<0.0001). 3088 females with data available at age 10 years were included in the model. Abbreviations: ART Antiretroviral therapy; BMIz Body Mass Index-for-age z-score; HAZ Height-for-age z-score; PI boosted protease inhibitor | | | | | | |

**Table S10:**  Multivariable analysis of association between characteristics at age 10 years and intensity of the pubertal growth in females

|  | Coef. | Standard error | t-value | p-value | Lower 95% CI | Upper 95% CI |
| --- | --- | --- | --- | --- | --- | --- |
| Region |  |  |  |  |  |  |
| East & Southern Africa |  |  |  |  |  |  |
| Botswana & South Africa | -0.207 | 0.059 | -3.51 | 0.000 | -0.322 | -0.091 |
| West & Central Africa | -0.187 | 0.102 | -1.83 | 0.068 | -0.388 | 0.014 |
| Europe & North America | -0.162 | 0.058 | -2.77 | 0.006 | -0.277 | -0.047 |
| Asia-Pacific | 0.075 | 0.078 | 0.96 | 0.337 | -0.078 | 0.228 |
| Central & South America, & the Caribbean | -0.251 | 0.097 | -2.6 | 0.009 | -0.441 | -0.062 |
|  |  |  |  |  |  |  |
| Initiated ART on PI based regimen | -0.018 | 0.016 | -1.07 | 0.284 | -0.050 | 0.015 |
|  |  |  |  |  |  |  |
| HAZ at age 10 years (FP comp 1) | -0.009 | 0.002 | -4.36 | 0.000 | -0.013 | -0.005 |
| HAZ at age 10 years (FP comp 2) | 0.142 | 0.020 | 6.98 | 0.000 | 0.102 | 0.181 |
|  |  |  |  |  |  |  |
| Age at ART initiation | 0.032 | 0.005 | 6.69 | 0.000 | 0.022 | 0.041 |
|  |  |  |  |  |  |  |
| BMIz at age 10 years (FP comp 1) | -0.001 | 0.000 | -3.74 | 0.000 | -0.002 | -0.001 |
| BMIz at age 10 years (FP comp 2) | 0.020 | 0.004 | 4.59 | 0.000 | 0.012 | 0.029 |
|  |  |  |  |  |  |  |
| Year of birth | -0.015 | 0.003 | -4.84 | 0.000 | -0.021 | -0.009 |
|  |  |  |  |  |  |  |
| Region X year of birth |  |  |  |  |  |  |
| Botswana & South Africa | 0.017 | 0.005 | 3.47 | 0.001 | 0.007 | 0.027 |
| West & Central Africa | 0.018 | 0.009 | 2 | 0.046 | 0.000 | 0.036 |
| Europe & North America | 0.018 | 0.005 | 3.4 | 0.001 | 0.008 | 0.028 |
| Asia-Pacific | 0.001 | 0.006 | 0.1 | 0.917 | -0.012 | 0.013 |
| Central & South America & the Caribbean | 0.027 | 0.008 | 3.21 | 0.001 | 0.010 | 0.043 |
|  |  |  |  |  |  |  |
| HAZ at age 10 ears X Age at ART initiation |  |  |  |  |  |  |
| HAZ FP Comp 1 X Age | 0.001 | 0.000 | 3.66 | 0.000 | 0.000 | 0.001 |
| HAZ FP Comp 2 X Age | -0.011 | 0.002 | -4.53 | 0.000 | -0.016 | -0.006 |
| A multivariable linear regression model was used to analyse factors associated with a parameter representing the intensity of the pubertal growth spurt estimated using SITAR. Lower values indicating lower intensity growth spurts and higher values indicating and more intense and rapid growth spurts. HAZ transformed as -HAZ/SD(HAZ), where SD(HAZ)=1.3) and BMIz (transformed as -BMIz/SD(BMIz), where SD(BMIz)=1.1) at age 10 years were modelled using a second order fractional polynomials (both FP(-1 -0.5)). Model includes interactions between region and year of birth (LRT, p=0.001) and age at ART initiation and HAZ at age 10 years (p<0.001). 3088 females with data available at age 10 years were included in the model. Abbreviations: ART Antiretroviral therapy; BMIz Body Mass Index-for-age z-score; HAZ Height-for-age z-score; PI boosted protease inhibitor | | | | | | |

**Table S11:** Multivariable analysis of association between characteristics at age 10 years and timing of the pubertal growth in males

|  | Coef. | Standard error | t-value | p-value | Lower 95% CI | Upper 95% CI |
| --- | --- | --- | --- | --- | --- | --- |
|  |  |  |  |  |  |  |
| Region |  |  |  |  |  |  |
| East & Southern Africa | -0.038 | 0.858 | -0.04 | 0.964 | -1.721 | 1.644 |
| Botswana & South Africa | -4.424 | 1.540 | -2.87 | 0.004 | -7.445 | -1.403 |
| West & Central Africa | -1.806 | 0.741 | -2.44 | 0.015 | -3.260 | -0.353 |
| Europe & North America | -2.909 | 1.035 | -2.81 | 0.005 | -4.939 | -0.879 |
| Asia-Pacific | -2.585 | 1.749 | -1.48 | 0.140 | -6.016 | 0.847 |
|  |  |  |  |  |  |  |
| Initiated ART on PI based regimen | -0.019 | 0.182 | -0.1 | 0.917 | -0.376 | 0.338 |
|  |  |  |  |  |  |  |
| HAZ at age 10 years (FP comp 1) | -0.433 | 0.133 | -3.26 | 0.001 | -0.693 | -0.173 |
| HAZ at age 10 years (FP comp 2) | -0.191 | 0.144 | -1.33 | 0.184 | -0.473 | 0.091 |
|  |  |  |  |  |  |  |
| Age at ART initiation (FP comp 1) | -0.001 | 0.000 | -3.24 | 0.001 | -0.001 | 0.000 |
|  |  |  |  |  |  |  |
| BMIz at age 10 years (FP comp 1) | 1.618 | 0.478 | 3.38 | 0.001 | 0.680 | 2.557 |
| BMIz at age 10 years (FP comp 2) | -0.896 | 0.265 | -3.38 | 0.001 | -1.416 | -0.376 |
|  |  |  |  |  |  |  |
| Year of birth | -0.144 | 0.059 | -2.46 | 0.014 | -0.259 | -0.029 |
|  |  |  |  |  |  |  |
| Region X year of birth |  |  |  |  |  |  |
| Botswana & South Africa | -0.003 | 0.080 | -0.04 | 0.970 | -0.159 | 0.153 |
| West & Central Africa | 0.457 | 0.154 | 2.96 | 0.003 | 0.155 | 0.760 |
| Europe & North America | 0.163 | 0.072 | 2.25 | 0.024 | 0.021 | 0.305 |
| Asia-Pacific | 0.224 | 0.096 | 2.32 | 0.020 | 0.035 | 0.413 |
| Central & South America & the Caribbean | 0.146 | 0.165 | 0.88 | 0.378 | -0.178 | 0.470 |
| A multivariable linear regression model was used to analyse factors associated with a parameter representing timing of the pubertal growth spurt estimated using SITAR. Coefficients represent differences in timing (in years) with lower values indicating earlier growth spurts and higher values indicating later growth spurs. Age, HAZ (transformed as -HAZ/SD(HAZ), where SD(HAZ)=1.3) and BMIz (transformed as -HAZ/SD(BMIz), where SD(BMIz)=1.1) at age 10 years were modelled using fractional polynomials (FP(3), FP(0 -0.5) and FP(0.5 1), respectively). Model includes interaction between region and year of birth (LRT, p=0.004). 1427 males with data available at age 10 years were included in the model. Abbreviations: ART Antiretroviral therapy; BMIz Body Mass Index-for-age z-score; HAZ Height-for-age z-score; PI boosted protease inhibitor | | | | | | |

**Table S12:** Multivariable analysis of association between characteristics at age 10 years and Intensity of the pubertal growth in males

|  | Coef. | Standard error | t-value | p-value | Lower 95% CI | Upper 95% CI |
| --- | --- | --- | --- | --- | --- | --- |
| Region |  |  |  |  |  |  |
| East & Southern Africa | ref |  |  |  |  |  |
| Botswana & South Africa | 0.021 | 0.014 | 1.58 | 0.115 | -0.005 | 0.048 |
| West & Central Africa | 0.009 | 0.020 | 0.45 | 0.655 | -0.030 | 0.048 |
| Europe & North America | 0.035 | 0.022 | 1.57 | 0.116 | -0.009 | 0.079 |
| Asia-Pacific | 0.147 | 0.016 | 8.9 | 0.000 | 0.114 | 0.179 |
| Central & South America, & the Caribbean | 0.085 | 0.028 | 3.1 | 0.002 | 0.031 | 0.139 |
|  |  |  |  |  |  |  |
| Initiated ART on PI based regimen | -0.010 | 0.018 | -0.56 | 0.577 | -0.044 | 0.025 |
|  |  |  |  |  |  |  |
| HAZ at age 10 years | 0.036 | 0.004 | 8.15 | 0.000 | 0.027 | 0.045 |
|  |  |  |  |  |  |  |
| Age at ART initiation | 0.004 | 0.003 | 1.13 | 0.258 | -0.003 | 0.010 |
|  |  |  |  |  |  |  |
| BMIz at age 10 year | 0.013 | 0.005 | 2.97 | 0.003 | 0.005 | 0.022 |
|  |  |  |  |  |  |  |
| Year of birth | -0.004 | 0.003 | -1.23 | 0.217 | -0.010 | 0.002 |
| A multivariable linear regression model was used to analyse factors associated with a parameter representing the intensity of the pubertal growth spurt estimated using SITAR. Lower values indicating less intense growth spurts and higher values indicating more intense and rapid growth spurts. 1427 males with data available at age 10 years were included in the model. Abbreviations: ART Antiretroviral therapy; BMIz Body Mass Index-for-age z-score; HAZ Height-for-age z-score; PI boosted protease inhibitor | | | | | | |
